# TRIM37 interacts with PTEN to promote the growth of human T-cell acute lymphocytic leukemia cells through regulating PI3K/AKT pathway

**DOI:** 10.3389/fonc.2022.1016725

**Published:** 2023-02-27

**Authors:** Honglan Qu, HASEN Gao-wa, Yanyan Hou, Mengwei Ren, Jun Li, Baoshong Jing, YanDan Du

**Affiliations:** Department of Hematology and Oncology, Inner Mongolia Forestry General Hospital, The Second Clinical Medical College of Inner Mongolia University for Nationalities, Yakeshi, China

**Keywords:** TRIM37, PI3K/AKT, PTEN, T-ALL, proliferation, apoptosis

## Abstract

**Background:**

TRIM37 has been reported to be associated with the tumorigenesis of cancers. However, the role of TRIM37 in T-cell acute lymphoblastic leukemia (T-ALL) remains unclear. This study aimed to characterize the effect of TRIM37 on T-ALL.

**Methods:**

TRIM37 expression in T-ALL patients and T-ALL cell lines was determined by qRT-PCR and Western blot. Knockdown or overexpression of TRIM37 was conducted by transferring small-interfering TRIM37 or lentivirus-mediated transducing into T-ALL cells. CCK-8 assay and flow cytometry assay were conducted to analyze the proliferation and apoptosis of T-ALL cells. Co-immunoprecipitation experiments were conducted to investigate the relationship between TRIM37 and PTEN and the ubiquitination of PTEN.

**Results:**

Our results suggested that TRIM37 expression was upregulated in the blood of T-ALL patients and T-ALL cell lines. Knockdown of TRIM37 noticeably inhibited the proliferation and promoted apoptosis of T-ALL cells. Ectopic expression of TRIM37 promoted the proliferation and suppressed the apoptosis rate of MOLT-4 cells and enhanced the phosphorylation of AKT. Moreover, TRIM37 interacted with PTEN and accelerated the degradation of PTEN *via* TRIM37-mediated ubiquitination in T-ALL cells. Moreover, TRIM37 reduced the sensitivity of T-ALL cells to bortezomib treatment. Additionally, PI3K/AKT signaling pathway was involved in the function of TRIM37 in T-ALL. TRIM37 contributed to the proliferation of T-ALL cells and reduced the susceptibility of T-ALL cells to bortezomib treatment through ubiquitination of PTEN and activating PI3K/AKT signaling pathway.

**Conclusions:**

Our study suggested that TRIM37 could be considered as a therapeutic target for T-ALL.

## Introduction

T-cell acute lymphoblastic leukemia (T-ALL) was derived from the malignant transformation of progenitor T cell infiltrating the bone marrow and peripheral blood. Clinically, T-ALL patients have exhibited typical symptoms including increased white blood cells count, hematopoietic failure, neutropenia, anemia, and thrombocytopenia (1). The occurrence of T-ALL is a multi-step process, resulting from abnormal genes that regulate cell growth, proliferation, survival, and differentiation. Among children and adult patients with ALL, T-ALL accounts for 15% and 25% of the cases, respectively (2, 3). Although intensive chemotherapy had improved the prognosis of T-ALL, the therapy strategy could not completely relieve the hematological tumor. Drug resistance and recurrence are the main factors affecting survival rates of T-ALL patients, but effective treatment for patients with relapse and poor prognosis still remains deficient (4, 5). Therefore, research efforts focusing on exploring the molecular mechanism of the development of T-ALL are urgently needed to search therapeutic targets and develop efficient and anti-leukemic drug with low toxic.

Tripartite motif (TRIM) protein family comprises more than 80 members in human and is found in all multicellular eukaryotes. Numerous evidences supported that TRIM family proteins participate in the occurrence and development of various diseases, namely, cancer, inflammation, pathogen infection, neuropsychiatric disorders, chromosomal abnormalities, and developmental diseases (1–3). Like the typical form of TRIM protein family members, TRIM37 consists of a RING finger domain at N-terminal, B-box domains, and a C-terminal coiled-coil (CO) domain (6, 7). The E3 ubiquitin ligase activity of RING domain can mediate the ubiquitination process of the target proteins by cooperating with the E2 ubiquitin binding enzyme (4). TRIM37 has been reported to be associated with the tumorigenesis and poor prognosis in multiple types of solid tumors. TRIM37 promotes transformation in breast cancer through acting as an H2A ubiquitin ligase by binding with PRC1 and PRC2 (8). High expressional level of TRIM37 resulted in enhanced proliferation, migration, and invasion of HCC cells (9), gastric cancer cells (10), and colorectal cancer (CRC) cells by epithelial-mesenchymal transition (EMT) stimulated by Wnt-catenin (11). TRIM37 contributes to the aggressiveness by activating NF-κB signaling pathway in non–small-cell lung cancer cells (12). Some TRIM family members such as TRIM31 (5), TRIM14 (13), TRIM65 (14), and TRIM22 (15) have been reported to participate in the progression of acute or chronic myeloid leukemia. We hypothesized that TRIM37 may play a role in the blood disease. After searching online, we found few studies about the TRIM37 in the blood disease. Therefore, we focused on the specific role and the regulatory mechanism of TRIM37 in T-ALL.

The phosphatidylinositol-3-kinase (PI3K)/Akt signaling pathway exerts critical role in a variety of steps of tumorigenesis. Activation of PI3K/AKT signaling pathway is commonly accompanied by AKT phosphorylation, which further stimulate AKT downstream target factors (16). Constitutively active PI3K/Akt signaling pathway is a common reason for abnormal cell proliferation and drug resistance of T-ALL and predicts a poorer prognosis of T-ALL patients (17). Recent studies demonstrated that TRIM37 markedly promoted metastasis of glioma cells and lung cancer cells through activating PI3K/Akt signaling pathway, implying a positive regulatory network between TRIM37 and PI3K/AKT in tumor (18, 19). Therefore, the proposed study aimed to investigate the role of TRIM37 in T-ALL and to explore the potential mechanism.

In the present study, we found TRIM37 was upregulated in T-ALL patients and T-ALL cell lines. Knockdown or overexpression of TRIM37 revealed the oncogenic role and bortezomib resistance effect of TRIM37 in T-ALL. Moreover, immunoprecipitation experiments proved that TRIM37 mediated the ubiquitination of PTEN, which led to PI3K/AKT signaling pathway activation in T-ALL cells.

## Materials and method

### Clinical samples

Human peripheral blood mononuclear cells were obtained from patients (*n* = 25) with T-ALL and normal individuals (*n* = 15) from The First Affiliated Hospital of Soochow University. All the participants signed the informed consent forms, and the project was approved by the Ethical and Scientific Committee of the Inner Mongolia Forestry General Hospital (20220816) and written informed consent was acquired from each participant.

### Cell culture and transfection

Human T-ALL cell lines, namely, Jurkat, MOLT-4, and RPMI8402 were brought from American Type Culture Collection (ATCC; Manassas, VA, USA) and cultured at 37°C in 5% CO_2_ in RPMI 1640 medium with 10% fetal bovine serum (FBS; Invitrogen, Carlsbad, CA, USA), 10 mM Hepes, and 100 U of penicillin/streptomycin. Human HEK 293T cells used for lentivirus package were maintained in Dulbecco’s Modified Eagle’s Medium (DMEM) supplemented with 10% FBS, 100 U of penicillin/streptomycin, 10 mM Hepes, and 2 mM L-glutamine (Invitrogen; Carlsbad, CA, USA) and were grown in humidified atmosphere with 5% CO_2_ atmosphere at 37°C.

Lipofectamine™ 2000 Transfection Reagent was used for plasmids transfection in HEK293T cells. Delivery of siRNA targeting TRIM37 and the negative control (si-NC) was conducted using Invitrogen™ DMRIE-C Transfection Reagent.

The plasmids of pLVX-TRIM37-Puro (Clontech, Mountain Vie, CA, USA) or empty vector control, together with psPAX2 and pMD2.G (Addgen, Watertown, MA, USA), were delivered into HKE293T cells by Lipofectamine™ 2000 (Invitrogen, Carlsbad, CA, USA) to generate lentivirus. Supernatants containing virus were harvested at 48h post-transfection. MOLT-4 cells were incubated with viral supernatants for 24h and then refreshed the supernatants with fresh medium supplemented with 0.25 μg/ml of puromycin for TRIM37-positive cell selection.

### Quantitative real-time PCR

Total RNA was isolated by TRIzol (Tiangen, Beijing, China), and the first strand of cDNA was synthesized with the PrimeScript RT reagent kit (Dalian, China) in accordance with manufacturer’s instruction. qPCR SYBR green real-time PCR master mix (TaKaRa, Dalian, China) was applied for PCR reactions run on ABI 7900HT sequence detection system. The relative mRNA expression was normalized to inner control GAPDH and was calculated by 2^−ΔΔCt^ method. The sequence of primers used for detecting TRIM37 is synthesized as follows: F: 5’-TGGACTTACTCGCAAATG-3’, R: 5’-ATCTGGTGGTGACAAATC-3’.

### Co-immunoprecipitation assay

MOLT-4 cells were lysed in RIPA buffer containing 50 mM pH 7.5 tris-HCl, 150 mM NaCl, 1% Nonidet P-40, 1 mM EDTA, 0.25% sodium deoxycholate, 1 mM Na3VO4, 1 mM NaF, 2% phenylmethylsulfonyl fluoride (Millipore, MA, USA) and protease inhibitor (Millipore, Bedford, MA, USA). Cell debris was eliminated after centrifuging at 10,000*g* at 4°C for 10 min. The obtained supernatants was transferred into fresh tubes for immunoprecipitation with 1 μg of indicated antibodies overnight at 4°C, followed by reaction with 12 μl of protein A/G Plus-agarose (Santa Cruz Biotechnology, Dallas, TX, USA) for 1h at 4°C. The immunocomplexes were then collected by centrifugation, washed by ice-cold RIPA buffer for five times and analyzed by Western blot experiments.

### Western blot assay

Proteins from the immunoprecipitate or cell lysates were separated by SDS-PAGE and then transferred onto polyvinylidene difluoride membranes (Millipore, Bedford, MA, USA) to incubate with primary antibodies incubation overnight at 4°C. After washing by 1× TBST buffer, the membrane was reacted with horseradish peroxidase (HRP) labeled-secondary antibodies for 1h at room temperature and protein bands were visually recorded.

### Cell proliferation assay

CCK-8 reagent (Beyotime, Shanghai, China) was adopted for detection following user’s manual. CCK-8 solution of 10 μl was added into cells seeded in 96-well plate at density of 6 × 10^3^ cells/well. Cells were harvested at indicated time points, and the cell proliferation was quantified after 2h incubation and was determined by the reading of OD450 on a microplate reader (Bio-Rad, Hercules, VT, USA).

### Cell apoptosis assay

Apoptosis of human T-ALL cell lines was determined using Annexin V-FITC/propidium iodide (AV/PI) Apoptosis Detection Kit (YEASEN, Shanghai, China). The experimental Jurkat and RPMI8402 cells were incubated with medium containing Annexin V-FITC and PI staining solution for 10 min without light. After adding 500 μl of 1× binding buffer, all the samples were gently mixed and placed on ice until flow cytometry detection performed by a FACS Calibur (BD Biosciences, San Jose, NJ, USA).

### Statistical analysis

All the experiments were performed at least three times. Data in the current study were exhibited as mean ± standard deviation (SD), represented three independent experiments with similar results. One-way ANOVA or a two-tailed Student’s *t*-test was used to calculate significant differences. The gray value of protein was determined by ImageJ software (National Institutes of Health, MD, USA). *P* value less than 0.05 indicated statistical difference.

## Results

### TRIM37 was upregulated in patients with T-ALL and T-ALL cells

**Figure 1:**
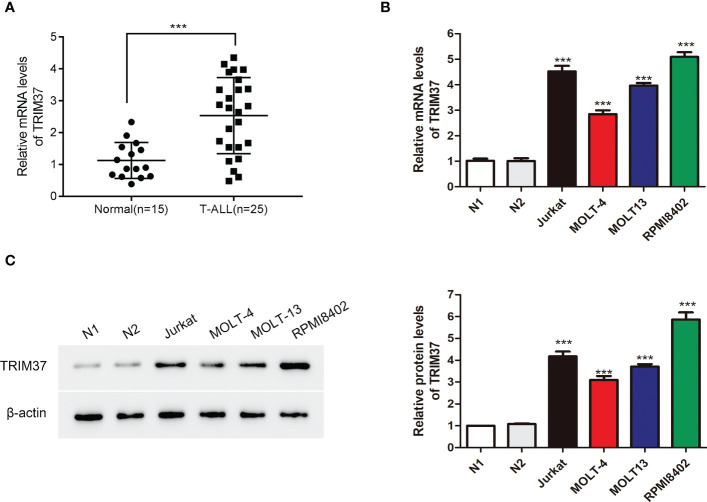
TRIM37 expression was elevated in patients with T-ALL and T-ALL cells. **(A)** The mRNA abundance of TRIM37 in T-ALL patients (*n* = 25) and normal individuals (*n* = 15) was examined by qRT-PCR. ***p < 0.001, compared with normal group. **(B, C)** The levels of TRIM37 in Jurkat, MOLT-4, MOLT13, RPMI8402, and two normal cell lines (N1 and N2) were quantified by qRT-PCR and Western blot assays. ***p < 0.001, compared with N1 group.

To investigate the function of TRIM37 in T-ALL, we firstly quantified the mRNA expression of TRIM37 in T-ALL patients and healthy individuals. Peripheral blood mononuclear cells from the patients with T-ALL and healthy donors were isolated, and qRT-PCR was conducted to analyze the mRNA level of TRIM37. Compared with control group, the expression of TRIM37 was significantly higher in patients with T-ALL (***P < 0.001, **Figure 1A**). Furthermore, the mRNA and protein expression levels of TRIM37 were observed in human T-ALL cells, namely, Jurkat, MOLT-4, MOLT13, and RPMI8402. Both the mRNA level and protein amount of TRIM37 were greatly upregulated in T-ALL cell lines compared with normal cells (***P < 0.001, **Figures 1B, C**). Based on the expression level of TRIM37 in different T-ALL cell lines, Jurkat and RPMI8402 cell lines were selected for knockdown studies and MOLT-4 cell line was selected for overexpression studies.

### Deficient expression of TRIM37 suppressed the proliferation of human T-ALL cells

**Figure 2:**
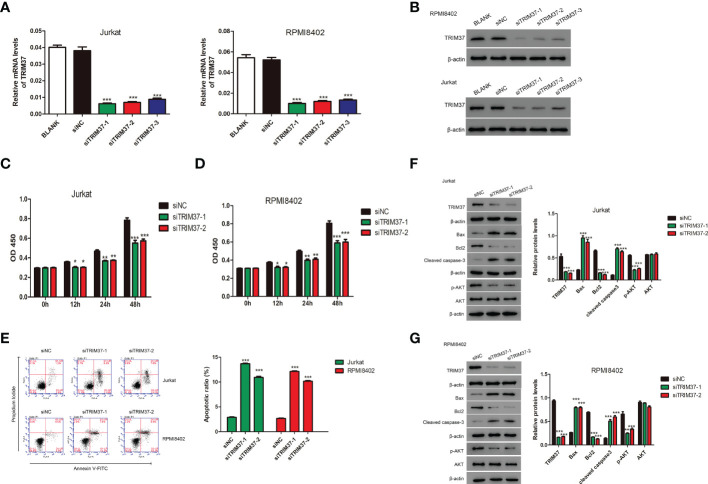
TRIM37 silencing suppressed the growth of human T-ALL cells. **(A, B)** The knockdown efficiency of siRNAs targeting TRIM37 was quantified by qRT-PCR and Western blotting. ***p < 0.001, compared with siNC group. **(C, D)** The CCK-8 assays for cell growth detection of Jurkat and RPMI8402 cells. *p < 0.05 compared with siNC group, **p < 0.01 compared with siNC group, ***p < 0.001 compared with siNC group. **(E)** Flow cytometry assays were performed to evaluate the apoptosis of Jurkat and RPMI8402 cells. ***p < 0.001, compared with siNC. **(F, G)** Western blotting was employed to examine the protein contents of TRIM37, Bax, Bcl2, cleaved caspase 3, p-AKT, and AKT in Jurkat and RPMI8402 cells transfected with/or without siTRIM37-1 or siTRIM37-2. ***p < 0.001, compared with siNC.

To deeply explore the effect of TRIM37 on the progression of T-ALL, three siRNAs targeting TRIM37 were chemically synthesized and delivered into Jurkat and RPMI8402 cells. The knockdown efficiency was evaluated by qRT-PCR and Western blot experiments. As presented in **Figures 2A, B**, the three siRNAs targeting TRIM37 effectively suppressed the mRNA and protein levels of TRIM37 (***P < 0.001). Cell proliferation assays revealed that knockdown of TRIM37 markedly repressed cell growth and promoted the apoptosis of Jurkat and RPMI8402 cells (*P < 0.05, **Figures 2C–E**). In addition, Western blot analysis showed that knockdown of TRIM37 improved the expression of apoptotic inducer Bax and apoptotic marker cleaved caspase 3 and decreased the abundance of anti-apoptotic molecule Bcl2 (**Figure 2F**). Interestingly, knockdown of TRIM37 had no effect on protein level of AKT, while obviously reduced the phosphorylation of AKT (**Figures 2F, G**).

### PI3K/AKT inhibitor LY294002 abolished the function of TRIM37 on cell proliferation in human MOLT-4 cells

**Figure 3:**
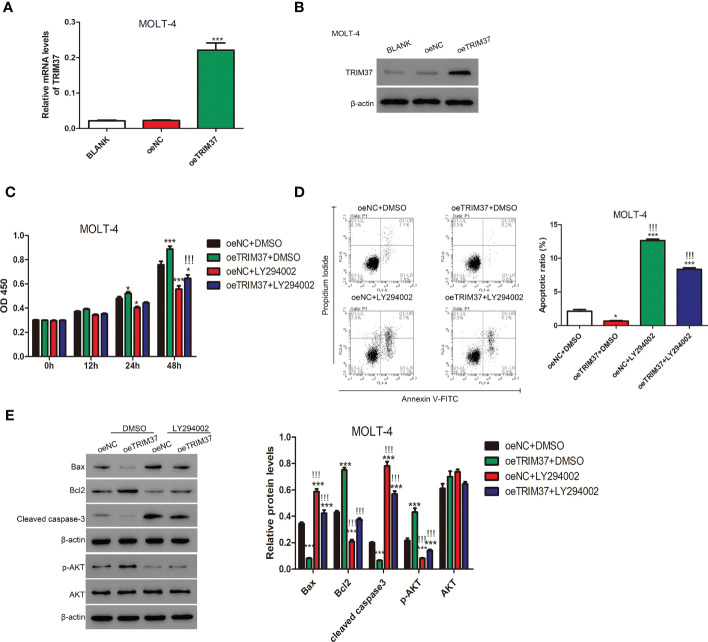
The PI3/AKT inhibitor LY294002 impaired the function of TRIM37 in human MOLT-4 cells. **(A, B)** The stable expression of TRIM37 in MOLT-4 cells was verified by qRT-PCR and Western blot assays. ***p < 0.001 compared with oeNC group. **(C)** The cell proliferation of MOLT-4 cells transduced with/or without trim37 was detected by CCK-8 assays. *p < 0.05, compared with oeNC + DMSO; ***p < 0.001 compared with oeNC + DMSO; !!!*p* < 0.001 compared with oeTRIM37 + DMSO. **(D)** The apoptosis of MOLT-4 cells transduced with/or without TRIM37 was detected by Flow cytometry. *p < 0.05 compared with oeNC + DMSO; ***p < 0.001 compared with oeNC + DMSO; !!!*p* < 0.001 compared with oeTRIM37 + DMSO. **(E)** Western blot experiments were exploited to examine the protein contents of Bax, Bcl2, cleaved caspase-3, p-AKT, and AKT in MOLT-4 cells transduced with/or without trim37. ***p < 0.001 compared with oeNC + DMSO, !!!*p* < 0.001 compared with oeTRIM37 + DMSO.

To further study whether TRIM37 regulate the proliferation of human T-ALL cells through AKT signaling pathway, MOLT-4 cell line stably expressing TRIM37 was established by lentivirus transfection. As shown in **Figures 3A, B**, the mRNA and protein expression of TRIM37 was significantly induced in oeTRIM37 group (***P < 0.001). CCK-8 assays revealed that overexpression of TRIM37 promoted cell growth of MOLT-4 cells (*P < 0.05). However, PI3K/AKT inhibitor LY294002 suppressed the cell growth of MOLT-4 cells at 24h post-treatment (**Figure 3C**). In addition, LY294002 treatment also significantly abolished the effect of TRIM37 on cell growth of MOLT-4 cells. As expected, overexpression of TRIM37 repressed the apoptotic rate of MOLT-4 cells, whereas inhibition of PI3K/AKT signaling pathway greatly promoted apoptosis of MOLT-4 cells transduced with TRIM37 (**Figure 3D**), which was further reflected by decreased amount of Bax and cleaved caspase 3 and upregulated Bcl2 (**Figure 3E**). In addition, ectopic expression of TRIM37 also increased the activation of PI3K/AKT signaling pathway, and the inhibitory effect of LY294002 on PI3K/AKT signaling were verified by Western blotting (**Figure 3E**).

### TRIM37 downregulated PTEN expression through ubiquitination

**Figure 4:**
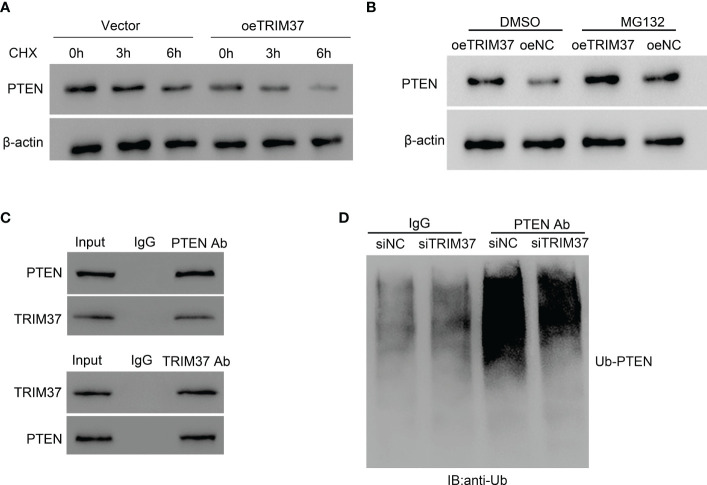
TRIM37 mediated the ubiquitination of PTEN in human MOLT-4 cells. **(A)** PTEN protein level was measured under 10 μM of CHX. **(B)** The effect of proteasome on PTEN expression was examined by Western blot assays. **(C)** Co-immunoprecipitation (Co-IP) experiments to investigate the interaction between TRIM37 and PTEN. Immunoprecipitation (Co-IP) experiments to detect the ubiquitination of PTEN. **(D)** Ubiquitination assay was used to examine the ubiquitination of PTEN in siNC and siTRIM37 transfecting cells.

PTEN is located upstream of the PI3K/AKT signaling pathway and can negatively regulate the PI3K/AKT signaling pathway. In the current study, we found that the expressional level of PTEN was negatively correlated with the abundance of TRIM37 in both T-ALL patients and normal individuals (**Supplementary Material Figure S1**). Because of the positive correlation between TRIM37 and PI3K/AKT signaling, we speculated that PTEN was involved in the TRIM37-PI3K/AKT regulatory axis. TRIM37 overexpression led to a rapid degradation of PTEN under CHX treatment (**Figure 4A**). However, addition of MG132, one proteasome inhibitor could prevent the loss of PTEN, implying the inhibition of PTEN depended on proteasome (**Figure 4B**). Furthermore, the interaction between TRIM37 and PTEN was determined by CO-IP. Endogenous TRIM37 and PTEN were pulled down by anti-PTEN and anti-TRIM37 antibodies, respectively. The interacting complex was detected by Western blotting. As shown in **Figure 4C**, TRIM37 specifically interacted with PTEN in MOLT-4 cells (IgG was used as negative control). Moreover, we immunoprecipitated the endogenous PTEN protein and conducted immunoblotting with anti-ubiquitin antibody. The results displayed that PTEN was ubiquitinated in the cells transfected with si-NC, whereas silence of TRIM37 significantly decreased the ubiquitination of PTEN, indicating TRIM37 could mediate ubiquitination of PTEN to decrease the expression of PTEN (**Figure 4D**).

### TRIM37 affected the therapeutic effect of Bortezomib on human T-ALL cells

**Figure 5:**
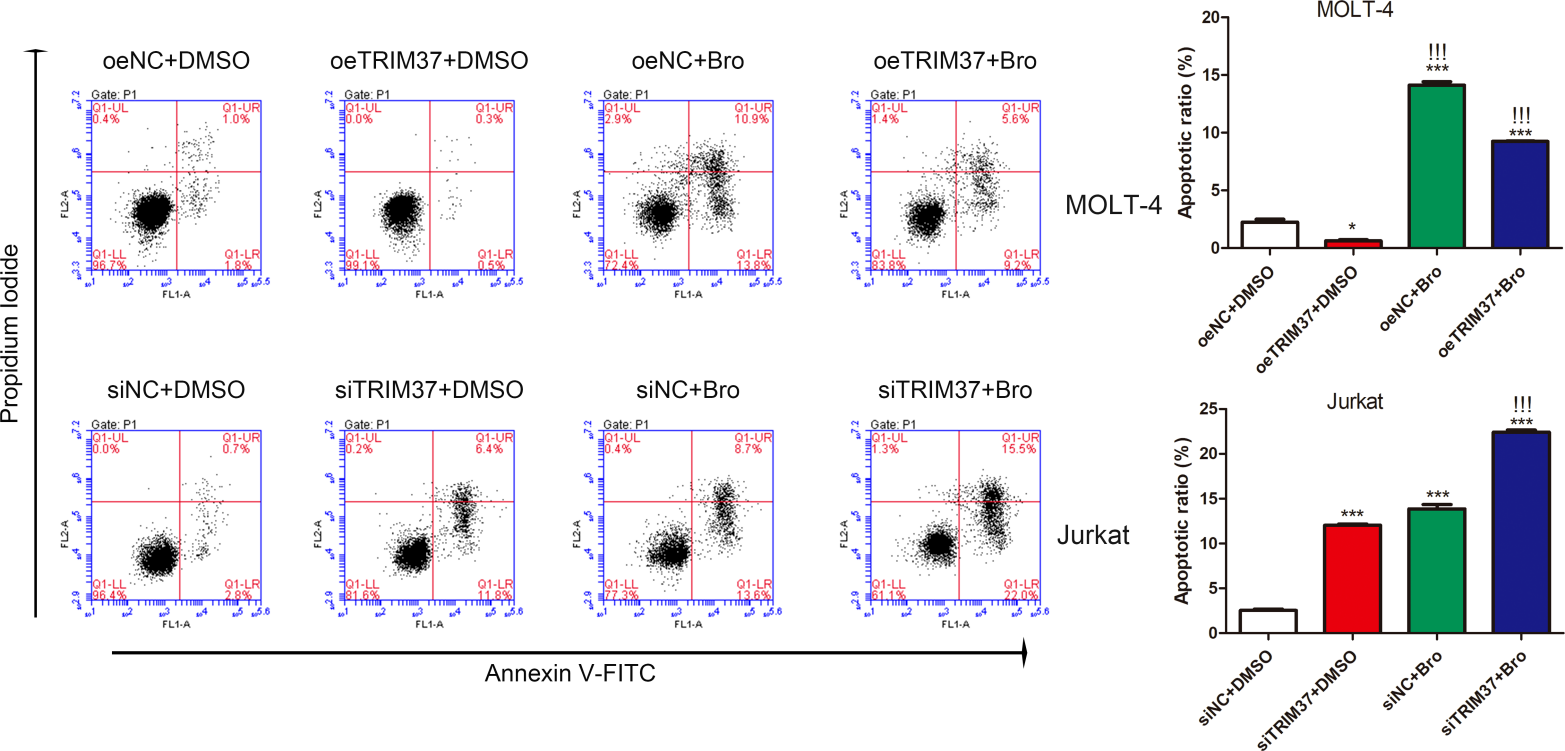
TRIM37 affected the therapeutic effect of Bortezomib on human T-ALL cells. *p < 0.05 compared with oeNC + DMSO; ***p < 0.001 compared with oeNC + DMSO; !!!*p* < 0.001 compared with oeTRIM37 + DMSO.

Given the fact that proteasome inhibitor bortezomib could sustain PTEN expression (Fujita et al., 2006), prompting us to hypothesize that TRIM37 may reduce the sensitivity of cells to bortezomib treatment through inhibiting PTEN. To verify the speculation, we performed FACS assays to investigate the co-effect of bortezomib and TRIM37 on apoptosis of human T-ALL cells. As shown in **Figure 5**, overexpression of TRIM37 significantly decreased cell apoptosis and impaired the effect of bortezomib on MOLI-4 cells. Inversely, knockdown of TRIM37 noticeably promoted cell apoptosis, and co-treatment of bortezomib greatly enhanced the effect of TRIM37 silence on mediating pro-apoptotic effect of Jurkat cells, which indicated that downregulated TRIM37 could improve the sensitivity of cells to bortezomib therapy.

## Discussion

The alteration of TRIM37 level contributed to the metastasis of several types of solid tumors and indicated the correlation between TRIM37 and poor prognosis of patients (8, 9, 11). However, the effect of TRIM37 in the progress of T-ALL has not been fully elucidated. In the current study, we firstly found that TRIM37 was increased in T-ALL patients and T-ALL cell lines. TRIM37 could promote the proliferation and repressed the apoptosis of T-ALL cells, indicating the oncogenic effect of TRIM37 on the development of T-ALL, and TRIM37 might be a potential therapeutic target for T-ALL clinical treatment.

Activation of PI3K leads to the production of phosphatidylinositol-3-phosphate (PIP3), which interacts with the PH domain of AKT to induce PDK1-mediated phosphorylation of AKT. The activated AKT further stimulates downstream target molecules to participate in regulation of various physiological processes (16). Excessive activation of the PI3K/AKT pathway is closely related to malignization of tumor (20–22) and is one of the most common reasons for the malignant development of T-ALL (23). In the present study, we proved that TRIM37 might positively regulated PI3K/AKT signaling pathway and our findings were consistent with the previous findings (18, 19). Moreover, aberrant PI3K/AKT signaling pathway counteracted the effect of TRIM37 on proliferation and metastasis of T-ALL cells (**Figure 3**), implying TRIM37 might promote the development of T-ALL through positively regulating of PI3K/AKT signaling pathway.

PTEN has been identified to negatively regulate the P13K/AKT signaling pathway by phosphorylating PIP3 to PIP2 (24). As one of the most vital tumor suppressors, the change of PTEN directly affected the occurrence, development, treatment, and prognosis of leukemia (25, 26). In T-ALL, high frequency of PTEN abnormalities, namely, mutation and inactive form can be commonly detected (27). Interestingly, Western blot experiments in clinical samples displayed that TRIM37 negatively correlated with the protein level of PTEN, suggesting that TRIM37 was a negative regulator for PTEN in T-ALL (**Supplementary Material Figure S1**). Post-translational modification is the major manner for modulating the intracellular PTEN (28). Previous study showed that K13 and K289 lysine sites on PTEN can be ubiquitinated to affect the stability and localization of PTEN (29), which is crucial for PI3K/AKT signaling pathway activation. TRIM37 decreased PTEN expression in a protease-dependent manner. Furthermore, TRIM37 directly interacted with PTEN to facilitate the ubiquitination modification on PTEN, which subsequently restored PI3K/AKT pathway activation. Our findings preliminarily defined a mechanism for PTEN regulation at the post-translational level in T-ALL cells, indicating that protease inhibitor can be considered as potential reagent in the combination therapy for T-ALL treatment. We could not eliminate RING domain-independent manner in the modulation of PTEN expression. The interaction domain between TRIM37 and PTEN remains to be studied to deeply understand the precise regulatory mode between TRIM37 and PTEN.

Ubiquitination plays an important role in cancer development and tumor metastasis through modulating the stability and functionality of oncoprotein and tumor suppressors (30). Recently, proteasome inhibitors have already been applied in clinical treatment of cancer (31). Bortezomib has been approved for clinical treatment of myeloma (MM), R/R MM, and mantle cell lymphoma (32) and can be combined with chemotherapy in acute lymphoblastic leukemia and lymphoma (33). The mechanism for bortezomib is that the boron atom in bortezomib has high affinity to the catalytic site of the 26S proteasome (34), so that bortezomib can possessed anti-tumor function as proteasome inhibitors (PIs) medication. Bortezomib could markedly restore PTEN level and elevate the drug sensitivity in trastuzumab-resistant cells (35–37). Combined with the results that TRIM37 mediated the ubiquitination of PTEN, we examined whether TRIM37 affected the effect of bortezomib on T-ALL cells growth. As expected, the effect of bortezomib on T-ALL cells apoptosis verified that TRIM37 promoted T-ALL cells proliferation depended on ubiquitination. In addition, knockdown of TRIM37 greatly boost the sensitivity of T-ALL cells to bortezomib (**Figure 5**). These results suggested decreased level of TRIM37 assist in elevating the efficacy of anti-tumor therapy, indicating that TRIM37 was one potential therapeutic target for clinical treatment of T-ALL. However, the insufficiency of the study is that we only conducted experiments *in vitro*. Both the survival analysis and the *in vivo* study of TRIM37 were required to further investigate the bio-function of TRIM37 in the malignization of T-ALL and the association between TRIM37 and the clinical prognosis of T-ALL patients.

## Conclusions

The proposed study revealed that TRIM37 was upregulated in T-ALL. TRIM37 promoted the proliferation of T-ALL cells and reduced the sensitivity to bortezomib of T-ALL cells at least in part through the excessive activation of PI3K/Akt signaling pathway and by ubiquitination of PTEN, suggesting TRIM37 might be used as therapeutic targets for T-ALL treatment.

## Data availability statement

The original contributions presented in the study are included in the article/**Supplementary material**. Further inquiries can be directed to the corresponding author.

## Ethics statement

In this study, the experiment involving human was permitted by the ethics committee of the Inner Mongolia Forestry General Hospital, Yakeshi city 022150, Inner Mongolia, China and written informed consent was acquired from each participates. The patients/participants provided their written informed consent to participate in this study.

## Author contributions

YD contributions to conception and design the project; HQ performed the experiment and wrote the draft; HG-w, YH, MR, JL, and BJ help to analyses the data and edit graph. All authors contributed to the article and approved the submitted version.

## Supplementary material

Supplementary Figure 1 TRIM37 was negatively correlated with PTEN in human T-ALL patients and normal people. ****p* < 0.001, compared with normal control group.
