# Supplementary figures and images for: TRIM37 interacts with PTEN to promote the growth of human T-cell acute lymphocytic leukemia cells through regulating PI3K/AKT pathway
Source: Front Oncol. 2023 Feb 27;12:1016725. doi: 10.3389/fonc.2022.1016725 (PMC10009101; doi:10.3389/fonc.2022.1016725)

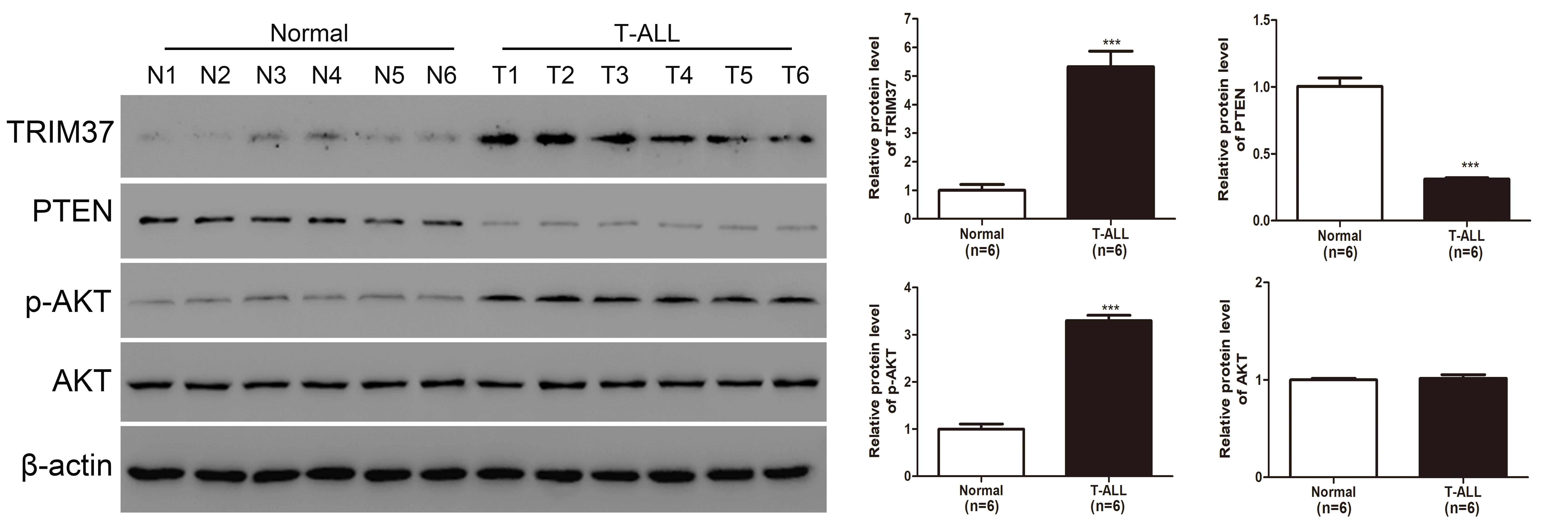

Supplement: Supplementary Figure 1 — TRIM37 was negatively correlated with PTEN in human T-ALL patients and normal people. ***p < 0.001, compared with normal control group. [file Image_1.tif]
